# Markers for internal neoplasia in the horse

**DOI:** 10.1002/vms3.1042

**Published:** 2022-12-10

**Authors:** Karolina Drozdzewska, Heidrun Gehlen

**Affiliations:** ^1^ Equine Clinic, Surgery and Radiology Freie Universitaet Berlin Berlin Germany

**Keywords:** equine, horse, marker, neoplasia, tumour

## Abstract

The diagnosis of internal neoplasia in horses is challenging. Increased production of hormones physiologic for adult animals (e.g., adrenocorticotropin, norepinephrine, and erythropoietin) or typical for the foetal phase (alpha‐fetoprotein, anti‐Müllerian hormone, and parathyroid‐hormone‐related protein) might aid in tumour diagnostics. Thymidine kinase‐1 and alkaline phosphatase are examples of intracellular enzymes, whose activity in the blood may increase in some neoplasia cases. Furthermore, inappropriate production of abnormal monoclonal or autologous antibodies can accompany lymphoma and multiple myeloma. Many of those tumour markers lead to clinical or laboratory changes, called paraneoplastic syndromes, such as hypercalcaemia and erythrocytosis.

The interpretation of the results of the tumour marker measurements in horses is complicated due to many factors affecting the markers’ concentration or activity (e.g., young age, pregnancy, and inflammation) and other diseases triggering the same changes. Moreover, the presence of paraneoplastic syndromes is inconsistent, which leads to low sensitivity of those substances as tumour markers.

In conclusion, screening for neoplasia in horses is not recommended. The measurement of tumour markers should be performed only in risk groups with suspicious clinical or laboratory findings, and the results should be interpreted with caution. It is advisable to add inflammatory markers to the tumour profile or repeat the measurements.

## Graphical abstract

The measurement of tumour markers in horses should be performed only in risk groups or in patients with suspicious clinical/laboratory findings (paraneoplastic syndromes). The results should be interpreted with caution as many factors (e.g., young age, pregnancy and inflammation) affect their concentration or activity. It is advisable to add inflammatory markers to the tumour profile or repeat the measurements. Abbreviations: ACTH, adrenocorticotropin; AFP, alpha‐fetoprotein; AMH, anti‐Müllerian hormone; EPO, erythropoietin; NE, norepinephrine; PTHrP, parathyroid‐hormone‐related protein.

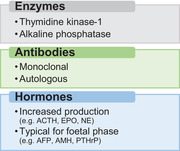

## 1 INTRODUCTION

The recognition of some integumentary neoplasia, such as melanoma, seems straightforward. Nevertheless, there is ongoing research on tests confirming the diagnosis in questionable cases or even predicting the development of some skin tumours, for example, a genetic test for a squamous cell carcinoma at the limbus in Haflingers (Lassaline et al., 2015) or a microRNA‐based test for sarcoids (Cosandey et al., 2021). However, the detection of internal neoplasia is a lot more challenging. First, the clinical symptoms are usually unspecific (e.g., weight loss and fever), and there are only a few predilecting factors (e.g., metastatic melanomas in grey horses). Standard workup depends on the suspected localisation of the tumour and includes blood work, diagnostic imaging of the thorax/abdomen, rectal palpation and endoscopic examination. The use of advanced imaging modalities, such as computed tomography, has also been reported recently (Fouche et al., 2016). If possible, a biopsy or fine needle aspiration can be performed and subsequently examined histopathologically. Alternatively, a peritoneal or pleural fluid can be examined cytologically, but both false‐positive and ‐negative results are possible (Recknagel et al., 2012).

A routine blood examination is rarely specific but might indicate organ dysfunction caused by a tumour (e.g., liver failure due to hepatocellular carcinoma) or pathologic cell production (e.g., lymphocytosis due to lymphoma). Moreover, neoplastic cells can produce or induce the production of specific substances that cause secondary changes in blood work or clinical signs, which are called paraneoplastic syndromes. Measuring the level of those circulating substances can help to diagnose, determine the stage or prognosis, monitor treatment and control for the recurrence of neoplasia; thus, they are called tumour markers. Paraneoplastic syndromes reported in horses include pruritis and alopecia, hypertrophic osteopathy (Marie's disease), pemphigus, hypoglycaemia, hypercalcaemia, erythrocytosis, neuropathies, immune‐mediated haemolytic anaemia (IMHA) or thrombocytopenia (IMTP), and monoclonal gammopathy (Axiak and Johnson, 2012; Möller, 2020). Only a few substances causing those changes are known, and even fewer can be measured in a horse. The aim of this review is to describe the circulating internal tumour markers available in equine medicine and the factors influencing their concentration or activity.

## 2 HORMONES

### 2.1 Adrenocorticotropin

Adrenocorticotropin (ACTH) is widely used as a marker of hormonally active adenoma or, less commonly, carcinoma of the pars intermedia of the pituitary gland in horses, which causes a clinical syndrome called pituitary pars intermedia dysfunction (PPID) (Equine Endocrinology Group, 2021; Heinrichs et al., 1990; Love, 1993). However, moderately higher levels of ACTH might also be caused by hyperplasia of the pars intermedia, and thus, this assay does not enable an exact diagnosis.

The pathognomonic clinical signs of PPID are hypertrichosis and a failure to shed the winter coat (McGowan et al., 2013b; Schott et al., 2017), but the risk of laminitis remains the most devastating complication of the disease (Karikoski et al., 2016). The lack of dopaminergic regulation from the hypothalamus results in the excessive production of proopiomelanocortin peptides, including ACTH, alpha‐melanocyte‐stimulating hormone, corticotropin‐like intermediate lobe peptide and beta‐endorphins by melanotropes. Measuring the alpha‐melanocyte‐stimulating hormone concentration was presumed to be more specific than ACTH for PPID diagnostic as the hormone is produced exclusively by melanotropes (ACTH can be produced by melanotropes and corticotropes), but it did not appear superior and is not commercially available (Beech et al., 2011b; McGowan et al., 2013a).

ACTH is released in the circadian rhythm in endocrinologically healthy horses, with the highest concentrations in the morning (Rendle et al., 2014). However, this fluctuation is absent in horses with PPID, and thus, the representative blood sample can be taken at any time of the day (Rendle et al., 2014). The ultradian rhythm is inconsistently detected, and a paired blood sample was not superior to the single timepoint ACTH measurement (Rendle et al., 2014). Nevertheless, the season strongly affects ACTH secretion, leading to the lowest concentrations in April (Durham, 2017) and the highest in late summer and autumn (Copas and Durham, 2012; Durham et al., 2021). The Equine Endocrinology Group (2021) recently recommended new reference values and cut‐off values for four different times of the year (Table 1). The likelihood of detecting disease in autumn is increased (McGowan et al., 2013a); however, there are challenges to this assessment because physiologic variability is also magnified (McFarlane, 2019). Grey horses, mares and thrifty breeds, including ponies and donkeys, have a significantly higher increase in ACTH concentration in September (Bamford et al., 2020; Durham and Shreeve, 2017; Fredrick et al., 2014; Potier and Durham, 2020). Furthermore, the age‐related rise in ACTH levels is highly pronounced in the autumn (Durham and Shreeve, 2017; McFarlane and Maxwell, 2017).

**TABLE 1:** Reported reference ranges and cut‐off values for selected tumour markers (the values presented should only be used as a guideline and the laboratory's own reference ranges are recommended)

Marker	Reference range	Cut‐off value	Literature
**ACTH**			
Dec–Jun		>40 pg/ml	Equine Endocrinology Group (2021)
July and Nov		>50 pg/ml	
Aug		>75 pg/ml	
Sep–Oct		>90 pg/ml	
10 min after TRH administration (Jan–Jun)		>200 pg/ml	
**AFP**			
	<10 ng/ml		Laboklin GmbH (2022)
**AMH**			
	<6.9 ng/ml		Renaudin et al. (2021)
		>10 ng/ml	Conley and Ball (2018)
**Testosterone**		>100 pg/ml	Conley and Ball (2018)
**Inhibine**		>0.8 ng/ml	Conley and Ball (2018)
**EPO**			
	8–25 mIU/ml		McFarlane et al. (1998)
	<9 mIU/ml		Jaussaud et al. (1994)
**NE**			
Calm	140–450 pg/ml		Yovich et al. (1984)
Excited	400–1200 pg/ml		
		>4× reference value	Fouche et al. (2016)
**PTHrP**			
	<1 pmol/L		Barton et al. (2004)
**ALP**			
	<450 IU/L		Laboklin GmbH (2022)
**TK1**			
	<2.2 U/L		Larsdotter et al. (2015)
	<1 pmol/min/ml		Wang et al. (2021)

Although animals with severe pain have higher plasma ACTH concentrations (Stewart et al., 2019), they remain stable in patients with chronic, mild‐to‐moderate pain (Gehlen et al., 2020), which enables diagnostic in horses presented with diseases such as laminitis (Gehlen et al., 2020). However, testing in a visibly excited or stressed animal (e.g., after transport or during bad weather) is not recommended, as it causes a temporary release of ACTH into the circulation (Chapman et al., 2017; Haffner et al., 2020). Sedation with alfa2 agonists and butorphanol does not influence the concentration of basal ACTH within the first 10 min (Oberhaus et al., 2021). Moreover, horses receiving grain have higher basal ACTH levels; thus, patients are allowed to eat only hay before testing (Diez de Castro et al., 2014; Jacob et al., 2018).

The appropriate management of blood samples is crucial to obtain correct results. The ethylenediamine tetraacetic acid blood should be constantly cooled and centrifuged within 2 h (Stewart et al., 2020). The concentration of ACTH in the separated plasma remains stable over the course of 48 h (Stewart et al., 2020). Freezing of the sample should be avoided because the ACTH concentration already drops after one freeze–thaw cycle (Hu et al., 2020). Furthermore, the assay for measuring ACTH may lead to significant differences, and new reference values given by the Equine Endocrinology Group (2021) reflect lower results obtained by chemiluminescence commonly used nowadays (McGilvray et al., 2020).

To sum up, the ACTH concentration is not recommended as a screening test to detect neoplasia of the pituitary pars intermedia, and the results should always be interpreted together with clinical signs, age, breed, colour, sex, feeding and health status and excitement of the horse, as well as the season, sample management and assay (Equine Endocrinology Group, 2021) (Table 2). Based on the studies available, the measurement of a basal ACTH concentration has an average sensitivity of 84% and specificity of 97% (Beech et al., 2011a; Beech et al., 2007; van der Kolk et al., 1995). The thyrotropin‐releasing hormone stimulation test with an ACTH measurement is more sensitive and enables the recognition of an early disease (Beech et al., 2011a; Beech et al., 2007; Beech et al., 2011b; Equine Endocrinology Group, 2021).

**TABLE 2:** Factors and diseases potentially affecting the activity or concentration of selected tumour markers

Marker	Tumour	Increase	Decrease/normal/absent
**Hormones**			
ACTH	Adenoma and adenocarcinoma of the pituitary pars intermedia	Hyperplasia of the pituitary pars intermedia, late summer and autumn, grey colour, ponies, age, stress, severe disease	Spring
AFP	Hepatoblastoma, hepatocellular carcinoma	Germ cell tumours, pregnancy, pregnancy abnormalities, foals, inflammation	Nonsecretory primary liver tumour
AMH	GCT	Intact males, other causes of ovary enlargement	Age >20 years (small number of antral follicles), early stage, nonsecretory tumour
EPO	EPO‐producing tumours of liver and metastatic carcinomas	Excitement, chronic hypoxia (high altitudes, lung/cardiac diseases), kidney disease, doping	Polycythaemia vera
NE, normetanephrin	Pheochromocytoma	Stress, excitement	Nonfunctional pheochromocytoma
PTHrP	Lymphoma, ameloblastoma, carcinoma, multiple myeloma	Pregnancy, lactation, intact males, idiopathic systemic granulomatous disease (sarcoidosis)	

**Enzymes**			
ALP	Osteosarcoma, liver tumours	Foals and young horses, intestinal disorders, cholestasis	
TK1	Lymphoma, multiple myeloma	Inflammation, viral infections, kidney insufficiency	

**Antibodies**			
Autologous Ig against erythrocytes/platelets	Lymphoma	Primary disease; secondary to bacterial or viral infection, drugs, toxins	
Monoclonal gammopathy	Multiple myeloma	Chronic inflammation on infection, chronic liver disease, lymphoma, other transient nonspecific antigenic stimulation	Nonsecretory multiple myeloma, early stage

### 2.2 Alpha‐fetoprotein

Alpha‐fetoprotein (AFP) is a glycoprotein produced by the yolk sac and later by the liver of the mammalian fetus (Gitlin and Boesman, 1967). It is widely used to detect pregnancy abnormalities in human medicine (Bader et al., 2004). There is also extensive research on its use in equine obstetrics‐the AFP level reaches a peak in the first trimester and decreases with the length of pregnancy (Vincze et al., 2015; Vincze et al., 2018). The AFP levels decline further postpartum over at least three weeks in healthy mares (Vincze et al., 2018) (Table 1) and over a minimum of one week in healthy foals (Prell, 2016).

The equine pregnancy abnormalities, including twinning, conception failure, placentitis and embryonic loss, cause increased AFP levels in the maternal serum (Canisso et al., 2015; Sorensen et al., 1990; Vincze et al., 2015; Vincze et al., 2018). Mizejewski (2015) discussed a possible role of human AFP as a positive inflammatory reactant in the prenatal phase or chronic viral hepatitis, and Prell (2016) reported significantly higher AFP levels in septic neonates in comparison to healthy ones.

Alongside the use of AFP in obstetrics, it is described as a liver tumour marker, as neoplastic cells originating from hepatocytes usually retain the ability to produce AFP (Tatarinov, 1964); thus, anti‐AFP antibodies can be used in immunohistochemical staining (Wu et al., 1997) and therapy after conjugation with cytotoxic drugs (Hata et al., 1984; Kato et al., 1983).

Nevertheless, not all primary liver tumours produce AFP; they do not secrete it into the circulation or release it in a pulsatile manner (Beeler‐Marfisi et al., 2010; Wu et al., 1997). Only 50 % of cases with hepatocellular carcinoma in human medicine have increased AFP levels in the blood (Bai et al., 2017; Song et al., 2016).

Primary liver tumours are extremely rare in equine medicine. Serum AFP was elevated in a case of hepatocellular carcinoma (Roby et al., 1990) and was within the reference range in a horse with hepatoblastoma (Beeler‐Marfisi et al., 2010). Additionally, the authors detected high blood AFP levels in an Arabian filly with hepatoblastoma confirmed post‐mortem. Unfortunately, little is known about the specificity of the marker, as it is not widely used in other hepatic diseases or inflammatory conditions. However, the authors’ personal observation was that the AFP level was not increased in four horses with presumed pyrrolizidine alkaloid intoxication, including two end‐stage liver failure cases.

The measurement of AFP is not recommended as a screening for liver tumours (Lennox et al., 2000), because many other factors, including individual variation, age, pregnancy, inflammation and other liver diseases, may change AFP levels in horses (Table 2). There are also reports of AFP‐producing tumours of germ cells (mainly the yolk sac, ovaries and testis), the urinary tract, the stomach, the bile duct and the pancreas in human (El‐Bahrawy, 2011).

Nevertheless, the AFP level can be a valuable adjacent diagnostic tool when suspecting a primary liver tumour in a young horse, as they were described only in the fetus (de Vries et al., 2013; Loynachan et al., 2007), neonates (Loynachan et al., 2007) and juvenile horses up to 4 years (Beeler‐Marfisi et al., 2010; Gold et al., 2008; Lennox et al., 2000; Tirosh‐Levy et al., 2019). The patients were usually diagnosed with liver dysfunction (Beeler‐Marfisi et al., 2010; Gold et al., 2008; Tirosh‐Levy et al., 2019) and were presented with ascites (de Vries et al., 2013; Loynachan et al., 2007) or pleural effusion (Axon et al., 2008), mucous congestion/erythrocytosis (Axon et al., 2008; Beeler‐Marfisi et al., 2010; Gold et al., 2008; Lennox et al., 2000; Roby et al., 1990; Tirosh‐Levy et al., 2019), persistent hypoglycaemia (Roby et al., 1990), pathologic fractures (Tirosh‐Levy et al., 2019), haemolysis and icterus (Beeler‐Marfisi et al., 2010).

### 2.3 Anti‐Müllerian hormone

Anti‐Müllerian hormone (AMH) and inhibin are protein hormones produced by Sertoli cells in the male fetus and play a role in sex differentiation (Conley and Ball, 2018). Testes in adult stallions are still able to produce AMH; thus, its level can be used to differentiate between intact males, including cryptorchid animals, and geldings (Claes et al., 2013). In mares, AMH and inhibin are produced by granulosa cells surrounding antral follicles and are widely used as markers of granulosa cell tumour (GCT), the most common ovarian neoplasia in horses (Ball et al., 2008; McCue, 1998). An enlargement and multicystic appearance (‘honeycomb’) of the affected ovary are typically present in an ultrasound examination (Renaudin et al., 2021; Sherlock et al., 2016). Most animals are presented with abdominal pain (Renaudin et al., 2021; Sherlock et al., 2016), behavioural changes (stallion‐like behaviour and aggression) and/or reproductive abnormalities (prolonged oestrus, nymphomania, and persistent anoestrus) resulting from disbalanced hormone production (Stabenfeldt et al., 1979). The most common of the latter are low serum progesterone concentrations (Ellenberger et al., 2007) and elevated levels of testosterone (40%–67% cases), inhibin (87%–90% cases) and AMH (Ball et al., 2013; Ball et al., 2008). The sensitivity of AMH depends on the cut‐off value and lies between 98% and 100% (Ball et al., 2013; Murase et al., 2018). It is superior to inhibin (sensitivity of 80%), testosterone (sensitivity of 48%) or even their combination (sensitivity of 84%) (Ball et al., 2013; Murase et al., 2018). Based on the large study by Uliani et al. (2019), the new upper reference range for AMH was raised from 4.7 (Murase et al., 2018) to 6.9 ng/ml (Renaudin et al., 2021). The accuracy of AMH is >90 %, with a cut‐off value of 10 ng/ml (Conley and Ball, 2018), owing to its sole production in the ovary, stability (long half‐life time‐36 h) (Conley and Ball, 2018) and unchanged concentration during the oestrus cycle or pregnancy (Conley and Ball, 2018; Uliani et al., 2019). However, the number of antral follicles and, thus, the age of the mare influence the serum AMH concentration, with variable values in young animals, high levels in mares aged 5–15 years and sinking concentrations after 20 years of age (Claes et al., 2015; Uliani et al., 2019). Additionally, increased AMH concentration was reported in other conditions causing transient enlargement of the ovary (e.g., haematoma) (Murase et al., 2018). Finally, false‐negative results are possible in endocrinologically inactive GCT or the early stages of the disease, and it is recommended to repeat the measurement after 2–3 months if symptoms persist as the tumour is expected to grow (Conley and Ball, 2018). Alternatively, an additional measurement of inhibin and testosterone levels can be performed as a panel of hormones, which slightly increases the sensitivity (Conley and Ball, 2018). However, both inhibin and testosterone secretion increase during ovulation and pregnancy (Conley and Ball, 2018; Ginther et al., 2007); thus, further assessment of the progesterone and/or estrone sulphate level might be advantageous in their interpretation (Conley and Ball, 2018). Inhibin is a lot less variable than steroid hormones, and its accuracy is higher than 80% with a cut‐off value of 0.8 ng/ml (Conley and Ball, 2018). Nevertheless, the specificity of the inhibin measurements was revealed to be low due to cross‐reaction with its precursors (Robertson et al., 1996). The assay detecting isoform B of inhibin proved to be superior in GCT diagnostic (Conley et al., 2018; Ellenberger et al., 2007) and is widely available now. Testosterone is a steroid hormone secreted by granulosa‐theca cells and by the adrenal cortex, which makes it the least accurate GCT marker, and only fairly high concentrations are likely to indicate GCT (Table 1) (Conley and Ball, 2018).

Complete excision of GCT can be confirmed endocrinologically because the hormone concentration drops to the reference range within a week (Conley and Ball, 2018).

### 2.4 Erythropoietin

The renal cortex in adult horses and the liver in fetus are the primary sites of erythropoietin (EPO) production (Jaussaud et al., 1994). Hypoxia triggers EPO release, which stimulates the proliferation and differentiation of erythrocytes in the bone marrow and increases haemoglobin synthesis (Jaussaud et al., 1994). It might be misused as a doping agent as it improves the performance of athletes (Jaussaud et al., 1994).

Highly increased EPO levels lead to absolute erythrocytosis and congestion of the mucous membranes, which can be a paraneoplastic syndrome (Axiak and Johnson, 2012). A relative erythrocytosis due to excitement (spleen contraction), dehydration or measurement error should be initially ruled out in all cases (Berlin, 1975; Murray, 1966; Tirosh‐Levy et al., 2019). The EPO levels were reported to be within normal limits or decreased in horses with primary erythrocytosis due to polycythemia vera, which can be confirmed by bone marrow aspiration (Beech et al., 1984; McFarlane et al., 1998; Munoz et al., 2009). Increased EPO concentrations (Table 1) might be physiologic (appropriate) or pathologic (inappropriate) (Axiak and Johnson, 2012; Berlin, 1975; McFarlane et al., 1998; Murray, 1966). Appropriate secondary erythrocytosis occurs due to EPO overproduction in patients with chronic tissue hypoxia (e.g., living at high altitudes, lung or cardiac diseases) (Tirosh‐Levy et al., 2019). EPO can also be inappropriately produced in diseased kidneys (e.g., hydronephrosis or polycystic kidney disease) or by specific tumours (McFarlane et al., 1998). Hepatocellular carcinoma (Roby et al., 1990), hepatoblastoma (Gold et al., 2008) and metastatic carcinoma (Cook et al., 1995) all producing EPO were described in horses. However, there are two case reports of equine hepatoblastoma with presumed secondary erythrocytosis without increased EPO concentration (Lennox et al., 2000; Tirosh‐Levy et al., 2019). The production of prostaglandin or another protein with EPO‐like action instead of the hormone itself was discussed as a possible mechanism (Cook et al., 1995; John et al., 1997). The protective and anti‐apoptotic roles of EPO on many cells, including neoplastic ones, were discovered recently and may explain the EPO production phenomenon in some tumours (Sytkowski, 2007).

The EPO seems a good marker, however, not a tumour‐specific marker, which can be used in cases of erythrocytosis after the exclusion of other possible causes (doping, excitement, hypoxia, polycythaemia vera and kidney diseases). Paraneoplastic secondary erythrocytosis seems more common than the primary one in horses (Beech et al., 1984; McFarlane et al., 1998).

### 2.5 Norepinephrine and normetanephrine

Pheochromocytoma is a rare adrenal medullary tumour arising from chromaffin cells able to secrete catecholamines (Luethy et al., 2016). It is usually benign, unilateral and asymptomatic (Luethy et al., 2016; Yovich et al., 1984); however, when hormonally active, it can lead to an adrenergic crisis and severe colic‐like signs, including tachycardia, tachypnoea, muscle tremor, sweating and anxiety (Luethy et al., 2016; Yovich et al., 1984). Hemoperitoneum after tumour rupture, myocardial lesions and multiple endocrine neoplasia has been described (Fouche et al., 2016; Luethy et al., 2016; Yovich et al., 1984). The most common clinicopathological findings are hyperglycaemia and hyperlactataemia (Fouche et al., 2016; Luethy et al., 2016; Yovich et al., 1984).

Yovich et al. (1984) described the measurement of blood norepinephrine as potentially diagnostic in horses, and the measurement of this hormone was the highest in the plasma and urine of a pony diagnosed with pheochromocytoma antemortem by Fouche et al. (2016). However, the measurement of adrenaline metabolites‐normetanephrine and metanephrine‐in plasma and urine is the test of choice in humans (Pappachan et al., 2014), and values higher than 4× the reference concentration are considered highly indicative of pheochromocytoma in people and dogs (Fouche et al., 2016; Salesov et al., 2015) (Table 1). Unfortunately, the reference range is not established in horses; thus, the results were compared to healthy controls, and only plasma normetanephrine and the urinary normetanephrine‐to‐creatinine ratio met these criteria in a clinical case (Fouche et al., 2016).

False‐negative results are possible in nonfunctional pheochromocytomas and with inappropriate handling of the samples (blood needs to be taken in a chilled heparin tube, protected from light, stored at −80°C after centrifugation and separation of plasma; additional acidification of the urine is necessary) (Fouche et al., 2016; Salesov et al., 2015). More research study and clinical reports are necessary to obtain reference and cut‐off values for pheochromocytoma diagnosis (Fouche et al., 2016).

### 2.6 Parathyroid‐hormone‐related protein

Parathyroid‐hormone‐related protein (PTHrP) plays a central role in calcium homeostasis and skeletal formation in the fetus, and its concentration drops considerably in adults (Wysolmerski and Stewart, 1998). However, it is also necessary for transplacental calcium transport and mammary development; thus, its blood level is increased in pregnant and lactating mammals (Wysolmerski, 2012). Nevertheless, PTHrP levels are the highest in the milk due to its local production in the mammary gland, and it is considered an important factor in promoting calcium secretion in the milk and intestinal calcium absorption in the foal (Care et al., 1997).

Suva et al. (1987) discovered that circulating PTHrP and calcium concentrations increase in mammals with malignant neoplasia (pseudoparathyroidism/humoral hypercalcemia of malignancy), and it was described in horses with lymphoma (Mair et al., 1990; Marr et al., 1989), multiple myeloma (Barton et al., 2004), ameloblastoma (Rosol et al., 1994), squamous cell carcinoma (Karcher et al., 1990; Meuten et al., 1978), adrenocortical (Fix and Miller, 1987) and metastatic carcinoma (Cook et al., 1995). The PTHrP shares the amino‐terminal region and acts through the same receptor as the prathyroid hormone, leading to excessive bone resorption, renal calcium reabsorption and phosphorus excretion (Suva et al., 1987). Neoplasia and humoral hypercalcemia of malignancy as a paraneoplastic syndrome should be considered in horses with hypercalcemia and hypophosphatemia after excluding renal disease (Toribio, 2011). Additional laboratory abnormalities associated with humoral hypercalcemia of malignancy include hypocalciuria, hyperphosphaturia, normal or decreased circulating PTH and increased PTHrP levels (Toribio, 2011). Reduced bone density and calcinosis (pathologic calcification of soft tissues) may occur over time (Toribio, 2011).

Human radioimmunoassay can be used to measure equine PTHrP in plasma or milk (Care et al., 1997; Ratcliffe et al., 1990), and the upper reference range is 1 pmol/L (Barton et al., 2004; Wysolmerski and Stewart, 1998) (Table 1). False‐negative results are possible if measuring PTHrP in order to diagnose malignancy, as few other mechanisms were reported to result in hypercalcemia in those cases, for example, the production of interleukins, tumour necrosis factor or bone destruction due to tumour invasion (Barton et al., 2004; Van der Kolk, 2007). The specificity of the test is high because pathological concentrations are found almost only in horses with malignancies; however, high circulating PTHrP was also described in a pony with idiopathic systemic granulomatous disease (sarcoidosis) (Sellers et al., 2001).

## 3 ENZYMES

### 3.1 Alkaline phosphatase

Alkaline phosphatase (ALP) is produced by hepatocytes, biliary epithelial cells, the intestine and osteoblasts; thus, an increase in its activity in the blood can be indicative of cholestasis, intestinal pathology or bone remodelling (Hank et al., 1993). High values are physiologic in neonates due to increased bone isoenzyme activity, and they decrease significantly over the first 21 days of life to normalise at the age of 2–4 years (Hank et al., 1993). Furthermore, bone tumours, mainly osteosarcomas in horses (Bush et al., 2007), lead to bone destruction and can cause a pathologic increase in ALP activity and hypercalcemia. Van der Kolk (2007) highlighted the importance of the differentiation between hypercalcemia of malignancy and those tumours. The specificity of total ALP activity as a marker for osteosarcoma is poor, but the measurement of a bone‐specific isoenzyme can increase it. Furthermore, ALP can be used as a prognostic marker (Jenner, 2010), and its activity should normalise after surgical excision of the osteosarcoma.

### 3.2 Thymidine kinase 1

Thymidine kinase 1 (TK1) is an intracellular enzyme that is activated in the G1 and S phases of the cell cycle when DNA doubles before mitosis or meiosis (Reichard and Estborn, 1951). There is a high production of this enzyme in fast‐proliferating neoplastic cells, and increased serum activity is caused by the leakage of TK1 after their death (Hallek et al., 1992); thus, the TK1 activity in serum correlates with the proliferative activity of the tumour (Hallek et al., 1992). However, the enzyme is highly unspecific as it can be released from many cells, including hepatocytes, hematopoietic and various neoplastic cells (Moore et al., 2021; Tanaka et al., 1993).

The serum activity of TK1 is high in the human fetus and remains elevated in maternal blood up to the 39th week of gestation (Bieniarz et al., 1988). Increased TK1 activity is also described in patients with vitamin B12 deficiency and kidney failure due to the impaired excretion of the enzyme with urine (Hagberg et al., 1984). The effects of age, pregnancy, vitamin B12 deficiency and kidney failure have not been studied in horses so far.

Furthermore, a few viral infections in humans, mainly caused by the family *Herpesviridae* (e.g., herpes simplex virus, Epstein‐Barr virus, varicella‐zoster virus and cytomegalovirus) and hepatotropic viruses, are associated with the increased activity of serum TK1 (Gronowitz et al., 1984; Tanaka et al., 1993). There are no reports of serum TK1 activity in horses with equine herpesvirus infection or acute viral hepatitis. A link between equine herpesvirus‐5 and lymphoma was described in a few case reports (Vander Werf and Davis, 2013; Vander Werf et al., 2014), and a potential cross‐reactivity between cellular (equine TK1) and viral (herpes) thymidine kinase should be further examined.

Inflammatory diseases cause a temporary elevation in TK1 activity in humans and dogs, most probably due to the proliferation of the leucocytes and their lysis at the site of inflammation (Gronowitz et al., 1983; Nakamura et al., 1997). A modest increase in the serum activity of TK1 was detected in a few horses with various inflammatory conditions (Larsdotter et al., 2015; Moeller et al., 2020; Moore et al., 2021).

A few reports of utilising TK1 as a biomarker of hematopoietic tumours in horses (lymphoma and multiple myeloma) were quite promising (Larsdotter et al., 2015; Moeller et al., 2020; Wang et al., 2021), but other studies showed that its performance is below expectations because false‐negative (Luethy et al., 2019; Moore et al., 2021) and false‐positive results are reported (Moeller et al., 2020). Moore et al. (2021) described no significant difference between the TK1 activity in equids with inflammatory disorders, hematopoietic and other neoplasia. On the other hand, Wang et al. (2021) found that TK1 performed well in distinguishing between horses with suspected/confirmed lymphoma and tumour‐free controls. Those discrepancies might be partially due to different assays used in both studies, with the method determining the TK1 activity using [^3^H]‐dThd as the substrate being more promising (Moore et al., 2021; Wang et al., 2021).

The reported sensitivity of TK1 as a tumour marker depends on the assay and corresponding cut‐off value (Table 1) and lies between 71% and 74% (Larsdotter et al., 2015; Wang et al., 2021). There is a huge discrepancy between the specificity reported to be 14% in one study (Larsdotter et al., 2015) and 97% in another (Wang et al., 2021). Nevertheless, using TK1 as a single tumour marker is not recommended, but a serial measurement of the enzyme might be superior (Moeller et al., 2020; Nakamura et al., 1997). It is described as a reliable monitoring factor (i.e., to assess the efficacy of the therapy or predict relapse) in dogs and people (Gronowitz et al., 1983; Nakamura et al., 1997). Interpreting the course of TK1 activity rather than a single measurement might also allow the differentiation from inflammation or viral infection, as it should return to normal limits as soon as the inflammation/infection has ceased (Larsdotter et al., 2015) and it should remain elevated in real neoplasia cases. Furthermore, interpretation of the results together with clinical findings and inflammatory markers (e.g., leucocyte count and serum amyloid A) might be useful (Larsdotter et al., 2015; Nakamura et al., 1997); however, an unspecific inflammatory reaction usually accompanies neoplasia (Winter et al., 2014).

## 4 ANTIBODIES/IMMUNOGLOBULINS

### 4.1 Autologous antibodies against erythrocytes and/or platelets

Both IMHA and IMTP were reported as a paraneoplastic syndrome in equine lymphoma cases (McGovern et al., 2011; Reef et al., 1984), and neoplasia is more common in horses with IMHA/IMTP than in healthy controls (Easton‐Jones et al., 2021). However, none of the 15 lymphoma cases described in the retrospective study by Luethy et al. (2019) had IMHA or IMTP.

Autologous antibodies can be detected by flow cytometry (Davis et al., 2002). Furthermore, immunoglobulins (Ig) against erythrocytes can be detected in the serum by an indirect Coombs test or on the erythrocytes’ surface by an direct Coombs test (Easton‐Jones et al., 2021). The results should be interpreted with caution because pathologic production of autologous Ig can be a primary or secondary condition triggered not only by neoplasia but also by drugs, toxins, bacterial (e.g., *Streptococcus* spp., *Clostridium* spp., and *Leptospira* spp.) or viral (equine infectious anaemia) infections (Easton‐Jones et al., 2021).

### 4.2 Monoclonal gammopathy

Multiple myeloma is a multifocal or diffuse malignant proliferation of plasma cells within the bone marrow or in extraosseous sites (e.g., lymph nodes, kidney, spleen) (Barton et al., 2004; Eberhardt et al., 2018; Edwards et al., 1993). A clone of Ig or its fragments (usually a light chain) is called a paraprotein or M‐component. They are produced in excess by neoplastic cells, causing hyperproteinaemia and a sharp peak in serum and/or urine electrophoresis (Eberhardt et al., 2018; Edwards et al., 1993). Monoclonal paraproteins can be detected in the alpha2, beta or gamma region, and they may belong to class IgG or IgA (Barton et al., 2004; Eberhardt et al., 2018; Edwards et al., 1993; Kent and Roberts, 1990).

The most consistent laboratory findings include hyperproteinaemia due to hyperglobulinaemia, hypoalbuminemia, anaemia and proteinuria (Eberhardt et al., 2018; Edwards et al., 1993); thus, multiple myeloma should be suspected in horses with those changes. If normal, serum electrophoresis should be repeated in suspicious cases as typical results might not be detected in the early course of the disease (Barton et al., 2004). Nevertheless, even serial tests might lead to false‐negative results in nonsecretory tumours (Edwards et al., 1993). Furthermore, chronic inflammation or infection, chronic liver disorders, other neoplasia and other antigenic stimulation lead to polyclonal gammopathy, which may complicate the diagnosis of a monoclonal increase of Ig (Barton et al., 2004; Eberhardt et al., 2018). Monoclonal gammopathies were also described in horses with lymphoma (Traub‐Dargatz et al., 1985) or with transient, benign disorders, which further highlighted the importance of repeated measurements (Kent and Roberts, 1990).

Radial immunodiffusion is another method allowing the classification of Ig and supporting the diagnosis of multiple myeloma in questionable cases (Barton et al., 2004). Bence‐Jones proteinuria can be present because overproduced light chains of Ig can be deposited in organs as AL‐amyloid (Kim et al., 2005) or secreted in the urine as Bence‐Jones bodies (Edwards et al., 1993). Eberhardt et al. (2018) recommended the human guidelines for the diagnostic of multiple myeloma in horses, which include detection of > 10% of plasma cells in a bone marrow biopsy or an extramedullary plasmacytoma and more than one of the following abnormalities: hypercalcaemia, kidney insufficiency, anaemia or bone lesions (Rajkumar et al., 2014).

## 5 SUMMARY

There are three main groups of internal tumour markers in equine medicine: hormones, enzymes (TK1, ALP) and antibodies (monoclonal gammopathy, autologous Ig against erythrocytes or thrombocytes). Unfortunately, none of the substances described is an ideal biomarker, and general screening for neoplasia in horses is not recommended. The results should be interpreted together with history, signalment, suspicious clinical signs or laboratory findings (i.e., paraneoplastic syndromes) and evaluated for consistency. Many factors increase the concentration/activity of the tumour markers reported (Table 2), and the most common are young age, pregnancy, lactation, inflammation, infection or severe disease. Interpretation of the results simultaneously with inflammatory markers can help to avoid false‐positive results, and tumour profiles usually contain them. Although inflammation often accompanies neoplasia, the values should normalise when the inflammation ceases, therefore serial measurements are recommended. Furthermore, tumours usually progress, and thus, the results of repeated tests remain elevated or increase.

More research needs to be undertaken to clarify the sensitivity and specificity of tumour markers in equine medicine.

## AUTHOR CONTRIBUTIONS

*Writing‐Original Draft Preparation*: Karolina Drozdzewska*. Writing‐Review and Editing*: Heidrun Gehlen.

## CONFLICT OF INTEREST

The authors state that they have no conflict of interest.

### ETHICS STATEMENT

The authors confirm that the ethical policies of the journal, as noted on the journal's author guidelines page, have been adhered to. No ethical approval was required as this is a review article with no original research data.

### PEER REVIEW

The peer review history for this article is available at https://publons.com/publon/10.1002/vms3.1042

## Data Availability

NA
